# Impact of lanadelumab in hereditary angioedema: a case series of 12 patients in Canada

**DOI:** 10.1186/s13223-021-00579-6

**Published:** 2021-07-23

**Authors:** Aled Iaboni, Amin Kanani, Gina Lacuesta, Christine Song, Manstein Kan, Stephen D. Betschel

**Affiliations:** 1 Allergy/Immunology Fellow, Department of Internal Medicine, Division of Clinical Immunology and Allergy, University of Toronto, St. Michael’s Hospital, Toronto, ON Canada; 2 Division of Allergy and Immunology, Department of Medicine, St. Paul’s Hospital, University of British Columbia, Vancouver, BC Canada; 3 Department of Medicine, Nova Scotia Health Authority, Dalhousie University, Halifax, NS Canada; 4 Department of Medicine, Division of Allergy and Immunology, St. Michael’s Hospital, University of Toronto, Toronto, ON Canada; 5 Division of Allergy and Immunology, Department of Medicine, University of British Columbia, Vancouver, BC Canada; 6 Medicine and Departmental Division Director for Clinical Immunology and Allergy, Division of Clinical Immunology and Allergy, University of Toronto, St. Michael’s Hospital, Toronto, ON Canada

**Keywords:** Lanadelumab, Hereditary angioedema, HAE, Swelling

## Abstract

**Background:**

Hereditary angioedema (HAE) is a rare autosomal dominant disease resulting in recurring episodes of swelling, leading to considerable patient morbidity and mortality. Lanadelumab is a plasma kallikrein inhibitor that is approved as 1st line therapy in Canada for long term prophylaxis of HAE attacks.

**Objective:**

To describe our clinical findings from a case series of adult patients with HAE type 1/2 who have been initiated on lanadelumab.

**Methods:**

A chart review of HAE type 1/2 patients at three academic centers in Canada was undertaken with demographic and clinical data extracted. Patients were included if they had been receiving lanadelumab for at least 6 months. Patients with other causes of angioedema were excluded.

**Results:**

12 patients meeting enrollment criteria were identified. Compared to pre-lanadelumab, patients had mean reductions of 72% and 62% in attack rate and treated attack rate respectively. 3 patients reported complete remission from attacks after starting lanadelumab. Most patients had significant improvements in HAE impact on social outings.

**Conclusion:**

Our case series findings support the 2019 International/Canadian HAE guideline that lanadelumab is an effective therapy for long term prophylaxis. In our patient population, initiation of lanadelumab improved disease control, minimized the burden of treatment and improved HAE impact on social outings.

## Background

Hereditary angioedema (HAE) is a rare genetic disease resulting in recurring episodes of painful swelling of the subcutaneous and submucosal tissue [1]. Attacks can affect many areas of the body. Most frequently involved are the extremities, gastrointestinal tract, genitals, face and upper airway. Swelling of the larynx can occur with potentially fatal consequences due to asphyxiation [2]. HAE patients with recurrent attacks have functional impairment, reduced quality of life (QoL) and increased mortality [3, 5]. The prevalence of HAE is approximately 1:50,000 individuals [3]. The condition is equally prevalent among sexes. Although quite variable, the mean age of onset for clinical symptoms is 11 years of age [4].

HAE is divided into 3 categories based on C1 esterase inhibitor enzyme (C1-INH) level and function. Patients who have quantitatively low levels of C1-INH are deemed to have type 1 HAE (HAE-1) and make up 85% of all cases. Those with a normal C1-INH antigenic level but dysfunctional enzyme activity are classified as type 2 HAE (HAE-2) and make up approximately 15% of cases [6]. HAE 1/2 result from mutations in the SERPING1 gene and are inherited in a predominantly autosomal dominant fashion [7]. A third category of HAE has normal C1-INH (HAE nC1-INH) and clinically presents similarly to HAE 1/2. HAE nC1-INH is exceedingly rare and the true prevalence is not known. Identified causes of HAE nC1-INH include genetic mutations in factor XII, angiopoietin 1, plasminogen, kininogen 1 and myoferlin [8]. In this case series, we will be addressing only patients with HAE 1/2.

In HAE, attacks of swelling are caused by overproduction of bradykinin that acts as a potent vasodilator. C1-INH inhibits various proteins along the bradykinin pathway including factor XIIa and plasma kallikrein. In HAE 1/2, C1-INH deficiency results in unopposed plasma kallikrein activity and overproduction of bradykinin. This overproduction causes vascular permeability and characteristic attacks of swelling [9].

Therapeutic options for HAE involve treatment of acute attacks, short term prophylaxis (STP), and long term prophylaxis (LTP). In Canada, licensed options available for treatment of acute attacks include icatibant, a bradykinin B2 receptor antagonist, and plasma-derived C1-INH (pdC1-INH). STP refers to the prophylactic treatment of HAE patients to reduce the risk of an attack during a high risk period such as dental surgery or upper airway manipulation. The treatment of choice for STP is intravenous (IV) pdC1-INH within an hour prior to the procedure. LTP is the use of ongoing, scheduled therapy to reduce attack frequency/severity and improve QoL in patients who are unable to meet their treatment goals with on-demand therapy alone. First line options for LTP in Canada include subcutaneous (SC) pdC1-INH and lanadelumab. Available second line options for LTP in some patients include attenuated androgens such as danazol and tranexamic acid (TXA), an antifibrinolytic [3].

Lanadelumab (Takhzyro™) is a fully humanized monoclonal antibody and is a highly potent and specific plasma kallikrein inhibitor, thereby reducing generation of bradykinin. It has a half-life of ~ 2 weeks and is given subcutaneously as a 300 mg injection once every 2 weeks, though this interval can be extended to 4 weeks if attacks are controlled for > 6 months [10, 11]. The HELP study was a Phase 3, randomized clinical trial (RCT) of Lanadelumab vs. Placebo involving 125 patients with HAE 1/2. The study showed that compared to placebo, lanadelumab significantly reduced attack frequency/severity and improved QoL over a 26 week treatment period [12]. The HELP study open-label extension (OLE) has since shown sustained reduction in attack frequency and a good drug safety profile [13]. Lanadelumab is now approved in Canada for use as LTP for HAE 1/2 in patients ≥ 12 years of age. Any patient ≥ 12 years of age with HAE ½ in Canada can be started on lanadeleumab for LTP. The decision to start lanadelumab is made jointly by the patient and treating physician and is based upon many factors including efficacy of on-demand therapy, frequency/severity of attacks and disease impact on QoL. It is not necessary to have failed other LTP agents before initiating lanadelumab [3].

To the best of our knowledge, there have been no reports outlining the clinical efficacy of lanadelumab in Canadian HAE patients. Herein, we describe our findings from a case series of 12 adult patients with HAE 1/2 who have been initiated on LTP with lanadelumab.

## Patients and methods

A retrospective chart review was conducted from three academic hospitals in Canada examining patients with HAE 1/2 who receive LTP with lanadelumab. The following inclusion criteria were used: Patients must have HAE 1/2 and be receiving lanadelumab for at least 6 months at the time of study enrollment. The diagnosis of HAE 1/2 must be established for enrolled patients based on a history of characteristic attacks of swelling and either low C1-INH level (Type 1) or low C1-INH function (Type 2). Patients with other etiologies of angioedema (histaminergic, acquired, or HAE nC1-INH) were excluded. While patient 10 (comorbid multiple myeloma) has not had acquired angioedema actively ruled out, her diagnosis of HAE 1 was established 18 years prior the onset of myeloma features. Patient 12 (comorbid T1DM) has not had acquired angioedema actively ruled out as his father also had HAE and died of upper airway angioedema, suggesting genetic inheritance. Enrollment for the study was completed March, 2021. All patients enrolled in the study were started on lanadelumab through compassionate initiation of the drug. Data was extracted from chart review and includes basic patient demographics and clinical information both before and after transition to lanadelumab.

A total of 12 patients were identified for this case series. From Toronto Ontario, Vancouver British Columbia, and Halifax Nova Scotia there were 7, 3, and 2 patients enrolled respectively. Demographic data are reported in Table 1. The mean age of enrolled patients is 43 years. The average age of HAE symptom onset was 18 years old. Excluding Patient 3, who was diagnosed prior to symptom onset due to an affected family member, the average latency from HAE symptom onset to diagnosis was 8 years. Most patients enrolled in the case series had few comorbidities. Patients 3 and 5 have a history of systemic lupus erythematosus and ulcerative colitis respectively (both quiescent), while Patient 10 is currently on maintenance therapy for Multiple Myeloma.

**Table 1:** Data of 11 HAE patients gathered from chart review including demographics, comorbidities, HAE type and time of diagnosis

Case	Age	Sex	HAE Type	Comorbidities	Other medications	Age symptom onset (years)	Age HAE diagnosis (years)
1	25	M	1	Nil	Nil	18	18
2	24	M	1	Nil	Nil	1	4
3	42	F	1	SLE (quiescent)	Nil	10	23
4	38	F	2	Nil	Nil	11	2
5	36	F	1	Ulcerative Colitis	Nil	4	12
6	30	F	1	GERD, Chronic Pain	Methadone	23	25
7	59	F	1	Nil	Nil	26	59
8	34	F	1	Depression	Citalopram	11	11
9	50	F	1	Nil	Nil	47	48
10	74	F	1	Multiple Myeloma, HTN	Revlimid, Telmisartan	Unknown	55
11	44	F	1	Raynaud’s, GERD, Cervical Stenosis	Esomeprazole	15	33
12	65	M	1	T1DM, Primary Adrenal Insufficiency	Insulin Pantoprazole	37	37

*GERD* gastroesophageal reflux disease, *SLE* systemic lupus erythematosus, *HTN* hypertension, *T1DM* type 1 diabetes

All clinical data were extracted from patient clinical encounter notes. Pre-lanadelumab clinical data were gathered from all notes in the year prior to initiating lanadelumab. Post-lanadelumab clinical data were gathered from the most recent 12 months of clinical notes. HAE impact on social outings (ISO) was gathered and coded by the treating MD on a scale of 1 to 5 (1 = no impact, 3 = some impact, 5 = a lot of impact) by MD interpretation of patient clinical encounter notes.

## Results

After a chart review of patient clinical encounter notes from pre- and post-lanadelumab, HAE disease manifestations and therapy history were extracted (Table 2). The type and prevalence HAE attacks experienced by patients were extremity swelling (9/12), abdominal pain (10/12), laryngeal swelling (7/12), lip swelling (3/12) and headaches (1/12). The attacks stated to be most debilitating were abdominal (8/12), laryngeal (4/12), and extremity swelling (1/11). Prior to initiating lanadelumab, most patients had received LTP with either IV or SC pdC1-INH (10/12). Patients 7 and 9 had never used LTP prior to initiating lanadelumab. All patients were started on lanadelumab between April 2019 and October 2020, which was made available for compassionate use, and remained on this therapy without interruption. The lanadelumab dosage used for 11/12 patients was 300 mg SC once every 2 weeks. Patient 4 had been switched from every 2 weeks to every 4 weeks in September, 2020 due to absence of attacks in the preceding 6 months. Acute treatment used by patients to treat breakthrough attacks was most commonly icatibant 30 mg SC.

**Table 2:** Clinical characteristics and HAE therapy history of case series patients

Case	Type of attacks	Most debilitating attacks	Pre-lana LTP	Pre-lana acute treatment	Lana transition date	Lana dosage	Post-lana acute treatment
1	Extremity swelling Abdominal pain Laryngeal swelling	Laryngeal swelling	C1-INH 2000U IV q3-4d	Icatibant 30 mg SC	January 2020	300 mg SC q2w	Icatibant 30 mg SC
2	Abdominal pain Lip swelling	Abdominal pain	C1-INH 3000U IV q3d	C1-INH 3000U IV	July 2019	300 mg SC q2w	C1-INH 3000U IV
3	Extremity swelling Laryngeal swelling	Laryngeal swelling	C1-INH 1500U IV q5d	Icatibant 30 mg SC	January 2020	300 mg SC q2w	Icatibant 30 mg SC
4	Extremity swelling Abdominal pain	Abdominal pain	C1-INH 1500U IV q3-4d	Icatibant 30 mg SC	October 2019	300 mg SC q4w	Icatibant 30 mg SC
5	Extremity swelling Abdominal pain Lip swelling Headaches	Abdominal pain	C1-INH 1500U IV q3-4d	C1-INH 3000U IV	April 2020	300 mg SC q2w	C1-INH 1500U IV
6	Abdominal pain Extremity swelling Laryngeal swelling	Abdominal pain	C1-INH 1500U IV q3d	Icatibant 30 mg SC	April 2019	300 mg SC q2w	Icatibant 30 mg SC
7	Abdominal pain Extremity swelling Laryngeal swelling	Abdominal pain	None	None	October 2020	300 mg SC q2w	Icatibant 30 mg SC
8	Abdominal pain Extremity swelling	Abdominal pain	C1-INH 4500U SC q3-4d	Icatibant 30 mg SC	February 2020	300 mg SC q2w	Icatibant 30 mg SC
9	Extremity swelling	Extremity swelling	None	Prednisone^a^	February 2020	300 mg SC q2w	Icatibant 30 mg SC
10	Abdominal pain Extremity swelling Laryngeal swelling	Abdominal attacks	Danazol C1-INH 4500U SC q3-4d	C1-INH IV	May 2019	300 mg SC q2w	C1-INH IV
11	Lip swelling Abdominal pain Laryngeal swelling	Laryngeal swelling	TXA C1-INH 1500U IV q2-3d	Icatibant or C1-INH IV	January 2020	300 mg SC q2w	Icatibant or C1-INH IV
12	Abdominal pain Laryngeal swelling	Abdominal, Laryngeal	Danazol C1-INH 2000U IV q3-4d	Icatibant or C1-INH IV	October 2020	300 mg SC q2w	Icatibant

All data was extracted from patient chart review

*LTP* long-term prophylaxis, *C1-INH* plasma-derived C1 esterase inhibitor enzyme, *TXA* tranexamic acid

^a^Prednisone was given to this patient as acute treatment prior to HAE diagnosis and was ineffective at treating attacks

Data from case series patients was extracted including HAE attack frequency, number of treated attacks, missed work days and HAE ISO (Table 3). The median number of attacks/year and treated attacks/year pre-lanadelumab was 11 (min = 3, max = 300) and 6 (min = 0, max = 50) respectively. The median HAE ISO pre-lanadelumab was 3 (min = 2, max = 5). There were 3 patients who had experienced missed work days as a result of HAE in the year prior to initiating lanadelumab.

**Table 3:** Case series data of the number of total/laryngeal HAE attacks in the year prior to initiating lanadelumab compared to the year following initiation of lanadelumab

Case	Pre-lanadelumab transition					Post-lanadelumab transition				
	Attacks/year	Laryngeal attacks/year	Treated attacks/year	Missed work days/year	ISO	Attacks /year	Laryngeal attacks/year	Treated attacks/year	Missed work days/year	ISO
1	10	4	6	6	2	2	0	2	0	1
2	12	0	6	20	2	4	0	0	0	1
3	8	2	8	0	4	2	1	2	0	2
4	6	0	6	0	3	0	0	0	0	1
5	50	0	25	0	5	20	0	20	0	2
6	300	50	50	Off^a^	5	100	40	40	Off^a^	1
7	15	0	0	Off^b^	4	12	0	0	Off^b^	1
8	6	0	6	0	3	2	0	2	0	2
9	10	0	6	0	2	0	0	0	0	1
10	30	0	12	Retired	3	6	0	6	Retired	1
11	12	0	12	Off^3^	2	6	0	6	Off^c^	1
12	3	0	3	Off^4^	3	0	0	0	Off^d^	3

Treated attacks are the number of times an acute attack was treated with breakthrough therapy. ISO was determined from MD review of patient clinical encounter notes pre- and post-lanadelumab. ISO, impact on social outings (1 = no impact, 3 = some, 5 = a lot)

^a^This patient is unable to work due to the frequency of her attacks

^b^This patient is a homemaker and is not employed

^c^This patient is off work due to a prior motor vehicle accident in 1999 causing cervical stenosis and progressive myelopathy

^d^This patient is off work due to chronic comorbid medical conditions (T1DM, Adrenal Insufficiency)

**Fig. 1:**
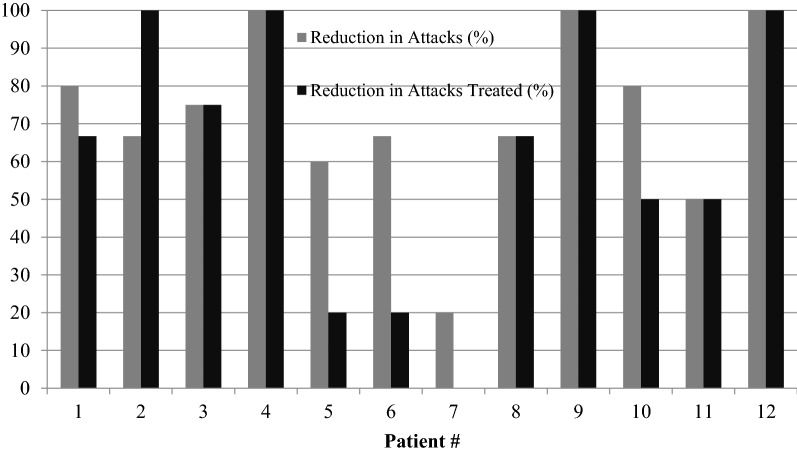
Percentage reduction in attack rate and treated attacks after initiating lanadelumab. Number of attacks and treated attacks were calculated using the total number from the year preceding and the year following initiation of lanadelumab

**Fig. 2:**
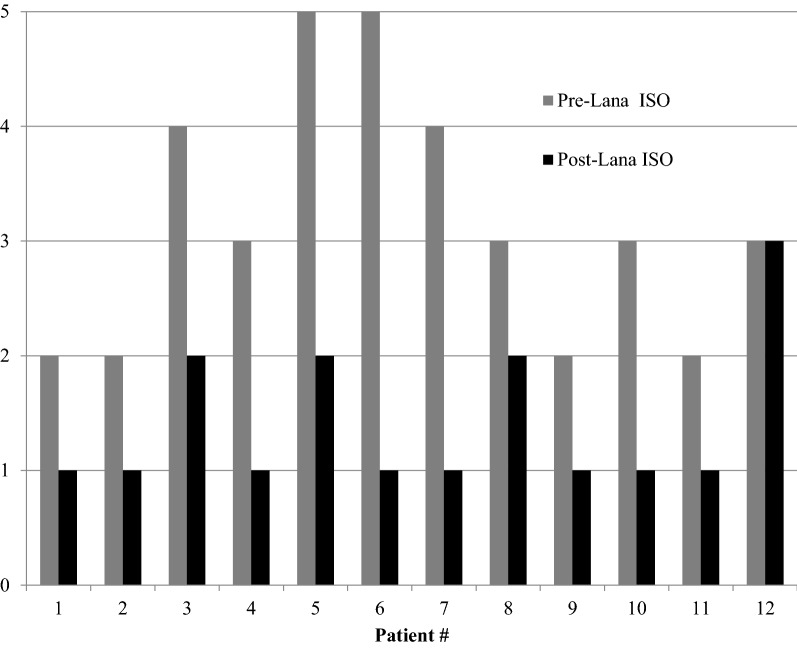
HAE impact on social outings (ISO) prior to and following initiation of lanadelumab. ISO was coded on a scale of 1 to 5 (1 = none, 3 = some, 5 = a lot). Pre-lanadelumab ISO was based on MD records prior to lanadelumab initiation. Post-lanadelumab ISO was based on MD records from the most recent patient encounter since starting lanadelumab

The median number of attacks/year and treated attacks/year post-lanadelumab was 3 (min = 0, max = 100) and 2 (min = 0, max = 40) respectively. Patients 4, 9 and 12 were completely free of attacks since initiating lanadelumab. The mean reduction in attack rate and treated attack rate post-lanadelumab transition was 72% and 62% respectively (Fig. 1). The median HAE ISO post-lanadelumab was 1 (min = 1, max = 3) with an average reduction of 1.8 compared to pre-lanadelumaab (Fig. 2). Post-lanadelumab, 8 out of 12 patients reported no social outing impact from their HAE. Only Patient 6 remained off work due to their HAE.

## Discussion and conclusions

Our case series demonstrates that LTP with lanadelumab resulted in considerable reductions in HAE attack rates in all patients enrolled in the study. Three of these patients had complete remission of HAE attacks. Most patients reported less impact of their HAE on social outings following initiation of therapy. It is notable that these improvements were seen in 10/12 patients who had been previously using LTP with pdC1-INH. The 2019 International/Canadian HAE guideline has recommended lanadelumab and SC pdC1-INH as 1^st^ line options for HAE-1/2 LTP (Consensus level of evidence) [3]. Our case series findings support this recommendation and show that lanadelumab is effective in the real-world Canadian HAE-1/2 patient population.

While not directly comparable, our study showed less efficacy of lanadelumab than the HELP study. Results from the HELP study show that HAE patients on lanadelumab 300 mg every 2 weeks had a mean attack rate reduction of 92.5% compared to the run-in period (0.26 vs. 3.5 attacks/month). 48.1% of patients on this dose were attack free [12]. This difference in results could be explained by the extensive use of pdC1-INH in our patient population. Only 40.7% of patients in the HELP study (lanadelumab 300 mg every 2 weeks arm) were on prophylaxis with pdC1-INH prior to enrolling in the study compared to 83% in our study. The increased use of LTP with pdC1-INH in our patient population prior to initiating lanadelumab likely resulted in better baseline disease control and reduced the magnitude of attack rate reduction after transitioning to lanadelumab.

Both IV and SC pdC1-INH used for LTP have been shown to reduce attack frequency, duration and severity in HAE 1/2 as seen in the CHANGE and COMPACT studies [14, 15]. To date, there have been no head-to-head trials comparing lanadelumab to these agents. A recent indirect treatment comparison study was done to compare LTP efficacy in the HELP trial (lanadelumab 300 mg SC every 2 or 4 weeks) and the CHANGE study (IV pdC1-INH 1000U every 3–4 days). The study showed a statistically significant reduction in HAE attack rate by 73% and 46% with lanadelumab q2w and q4w compared to IV pdC1-INH [16]. In line with findings from this indirect treatment comparison, 9/12 of our case series patients previously used IV C1-INH prior to switching to lanadelumab and all had significant reductions in attack rates after this switch. Our cases and this indirect treatment comparison provide evidence that lanadelumab results in potentially improved efficacy when used as LTP compared to IV pdC1-INH, though a head to head trial would be required to confirm this. An ITC has not yet been performed between the HELP and COMPACT studies but would be useful to compare efficacy between lanadelumab and SC pdC1-INH.

In addition to the suspected advantage in efficacy over IV pdC1-INH, lanadelumab is also easier to self-administer through the SC route which is likely to improve compliance. An interim analysis from the HELP study OLE showed that 70.9% of patients preferred the SC route of administration compared to IV pdC1-INH [17]. Compared to SC pdC1-INH that is dosed every 3–4 days, lanadelumab is more convenient to administer as it is dosed every 2–4 weeks.

Overall, our case series supports the existing 2019 International/Canadian HAE guideline that lanadelumab is effective when used as LTP in reducing patient attack rates, ISO and missed work days in the Canadian HAE 1/2 population. Our findings suggest that patients with HAE refractory to LTP with IV pdC1-INH, and possibly SC pdC1-INH, could consider switching to lanadelumab to attain better disease control and minimize the burden of treatment. A head-to-head comparison between these agents would be beneficial to support this recommendation. A possible limitation of our study is that all patients received lanadelumab through compassionate access, which could lead to them perceiving and reporting better disease control while on the drug. Future areas that remain to be explored include the role lanadelumab as LTP in acquired angioedema and HAE nC1-INH.

## Data Availability

All data collected for the purposes of this study comply with field standards. The datasets used and/or analyzed during the current study are available from the corresponding author on reasonable request.

## Abbreviations

HAE: Hereditary angioedema
QoL: Quality of life
C1-INH: C1 esterase inhibitor enzyme
HAE-1: Type 1 hereditary angioedema
HAE-2: Type 2 hereditary angioedema
HAE nC1-INH: Hereditary angioedema with normal C1 esterase inhibitor enzyme
STP: Short term prophylaxis
LTP: Long term prophylaxis
pdC1-INH: Plasma-derived C1-INH
IV: Intravenous
SC: Subcutaneous
TXA: Tranexamic acid
RCT: Randomized clinical trial
SLE: Systemic lupus erythematosus
GERD: Gastroesophageal reflux disease
HTN: Hypertension
ISO: Impact on social outings
ITC: Indirect treatment comparison
OLE: Open label extension
